# Sustaining the Board of Regents

**Published:** 1868-07

**Authors:** 


SUSTAINING THE BOARD OF REGENTS.—The Am. Institute of Homœopathy,

*Resolved*. That the action of the Board of Regents in appointing Prof. C. J. Hempel to the chair of Homœopathy in the University of Michigan, receives the hearty and unqualified approval of this Institute.

*Resolved*, That should any or all of the Allopathic chairs of the medical department of said University be vacated, and the Board of Regents see fit to appoint Homœopathic medical men to fill these chairs, the Homœopathic profession of America will pledge its influence to the medical department of that University in sustaining such action.

